# An Adaptive Filter for Nonlinear Multi-Sensor Systems with Heavy-Tailed Noise

**DOI:** 10.3390/s20236757

**Published:** 2020-11-26

**Authors:** Xiangxiang Dong, Luigi Chisci, Yunze Cai

**Affiliations:** 1 Department of Automation, Shanghai Jiao Tong University, Shanghai 200240, China; js.danesir@sjtu.edu.cn; 2 Key Laboratory of System Control and Information Processing, Ministry of Education of China, Shanghai 200240, China; 3 Key Laboratory of Marine Intelligent Equipment and System of Ministry of Education, Shanghai Jiao Tong University, Shanghai 200240, China; 4 Department of Information Engineering, Università di Firenze, 50139 Firenze, Italy; luigi.chisci@unifi.it

**Keywords:** nonlinear multi-sensor system, heavy-tailed noise, student’s t distribution, spherical-radial cubature rule, information fusion

## Abstract

Aiming towards state estimation and information fusion for nonlinear systems with heavy-tailed measurement noise, a variational Bayesian Student’s t-based cubature information filter (VBST-CIF) is designed. Furthermore, a multi-sensor variational Bayesian Student’s t-based cubature information feedback fusion (VBST-CIFF) algorithm is also derived. In the proposed VBST-CIF, the spherical-radial cubature (SRC) rule is embedded into the variational Bayes (VB) method for a joint estimation of states and scale matrix, degree-of-freedom (DOF) parameter, as well as an auxiliary parameter in the nonlinear system with heavy-tailed noise. The designed VBST-CIF facilitates multi-sensor fusion, allowing to derive a VBST-CIFF algorithm based on multi-sensor information feedback fusion. The performance of the proposed algorithms is assessed in target tracking scenarios. Simulation results demonstrate that the proposed VBST-CIF/VBST-CIFF outperform the conventional cubature information filter (CIF) and cubature information feedback fusion (CIFF) algorithms.

## 1. Introduction

The Kalman filter (KF) is an optimal state estimator for linear state-space systems [1,2,3]. It is widely used, owing to its optimality, in many applications like, e.g.,  localization, control, target tracking, and signal processing [4,5,6,7,8,9,10,11,12,13,14,15,16,17]. In reality, however, systems are usually characterized by strong non-linearities which make the conventional KF inappropriate. To this end, nonlinear filtering methods have been developed like, e.g., function approximation, deterministic sampling, and Monte Carlo estimation methods [18,19,20]. The function approximation method adopted by the extended Kalman filter (EKF) approximates the nonlinear system equations through truncated Taylor expansions [21]. However, the Jacobian matrix is not computable for systems with non-smooth non-linearities [21]. As for the deterministic sampling method, its main representatives are the unscented Kalman filter (UKF), cubature Kalman filter (CKF), etc. [22,23]. Unfortunately, the state error covariance matrix (SECM) in UKF may result non-positive definite for high-dimensional systems, possibly leading to filter divergence [23]. To overcome the drawbacks of UKF, Arasaratnam and Haykin proposed CKF in 2009 [23]. CKF approximates the posterior probability density function (PDF) by means of the spherical-radial cubature (SRC) rule, thus resulting in improved filtering accuracy for high-dimensional systems [24]. Moreover, compared to the Monte Carlo estimation approach exploited by particle filters (PFs), CKF is not affected by particle depletion issues.

Recall that the KF (and also EKF, UKF, and CKF) can also be implemented in the alternative information filter (IF) form wherein the inverse covariance (information) matrix is propagated instead of the covariance. In this respect, the IF form greatly simplifies the measurement update compared to the traditional covariance form [25]. In this way, it facilitates multi-sensor fusion, wherein measurements from different sensors are fused via directly adding information contributions to the information matrix and vector [26]. Considering the above-stated superiority in the multi-sensor case, the cubature information filter (CIF) will be adopted as a filtering approach in this paper.

Unfortunately, the existing CIF assumes the known and constant measurement noise covariance matrix (MNCM) [27]. However, due to the complex environment where the sensor is located, it is prone to measurement outliers. This might induce a heavy-tailed measurement noise [28]. Actually, an outlier is characterized by the abnormality of its measurement value; correspondingly, outliers might give rise to a heavy-tailed distribution of the measurement noise. There are many practical applications where there is heavy-tailed noise [29,30,31,32]. For instance, in vision-aided inertial navigation, either computer vision data contaminated by outliers or sonar data corrupted by phase noise may lead to heavy-tailed noise [29]. In the detection of FM signals, heavy-tailed noise can be produced by thunderstorms or an iceberg breakup in under-ice acoustics [30]. In target tracking scenarios, measurement outliers come from unreliable sensors and/or targets [31], while in target detection with synthetic aperture radar, heavy-tailed noise occurs due to clutter and buried or obscured observed targets [32], etc. Compared to Gaussian noise (hypothesized by conventional CIF), heavy-tailed noise is characterized by greater uncertainty in its distribution tails, yielding the heavy-tailed shape of the PDF [33]. Under the above circumstance, the filtering accuracy of CIF may deteriorate and filter divergence may occur, due to the discrepancy between the assumed noise statistics and the true one.

To tackle the filtering problem under heavy-tailed noise, several adaptive methods have been developed, including generalized maximum likelihood [34], multi-model [35], and joint estimation [36] methods. Specifically, a representative of generalized maximum likelihood estimation is the Huber-based filter, which minimizes the combined $l_{1}$ and $l_{2}$ norms [37] and, however, leads to limited estimation performance [38]. An alternative is the multi-model approach, which models the unknown noise as a random parameter switching according to a Markov Chain and is able to adaptively estimate the system state [35]. Unfortunately, it is limited by the selected sets of models. The greater is the deviation between the selected models and real noise model, the worse the estimation performance of this method [36]. Furthermore, its computational complexity will increase significantly as the number of unknown parameters and selected models increases. The joint estimation method includes expectation-maximization (EM) and variational Bayes (VB) approaches [36]. The EM approach identifies and estimates system states as well as hidden variables through expectation (E) and maximization (M) steps. However, it is difficult to obtain the analytical solution for E and M estimates in the case of high-dimensional systems.

Conversely, another joint estimation method, the VB approach, can avoid the aforementioned drawbacks of the EM approach for high-dimensional systems. By searching an approximation for the true joint distribution, the VB approach is widely used in the adaptive joint estimation of noise statistics [27,29,38,39,40,41,42]. Agamennoni et al. proposed a robust Kalman filter by exploiting a flexible model [39] and also introduced a structured variational approximation approach for heavy-tailed noise [40]. However, the use of these filters is restricted to linear systems. Huang et al. [38] also presented a linear Student’s t (ST) filter. By modeling heavy-tailed noise with ST distribution, the system state as well as noise statistics are adaptively estimated through VB iterations. Unfortunately, this approach is only applicable to linear systems, too. Although there has been some research work on nonlinear systems in [29,41,42], the proposed filters rely on the covariance filter form, instead of the IF form, in order to jointly estimate system state, SECM, and noise statistics, thus resulting into complex computation. Moreover, the current VB approach cannot be embedded into nonlinear CIF directly, since the computation of noise parameters like the scale matrix involves a nonlinear function and, therefore, requires the use of the SRC rule. Hence, to jointly estimate states and parameters of the noise statistics (especially the scale matrix) via the information filter framework is still an open problem in nonlinear systems with heavy-tailed noise. In addition, the existing research work is only able to deal with single-sensor estimation problems. For nonlinear multi-sensor systems with heavy-tailed noise, the current state-of-art does not allow to estimate and fuse the states as well as noise statistics.

To conclude, there still exist the following issues to be addressed for the state estimation of nonlinear multi-sensor systems. On the one hand, the nonlinear information filter cannot be embedded into the current VB framework, since the scale matrix cannot be directly computed in the same way as for linear or nonlinear systems in the covariance filter form. On the other hand, it is not suited to multi-sensor systems. There are different heavy-tailed noise signals when multiple sensors are working in a multi-sensor system. This makes the conventional filter for the single-sensor case fail to fuse different estimated states. Thus, for nonlinear systems with heavy-tailed noise, especially systems with multiple sensors, it is still an open issue how to jointly estimate system state and noise statistics by combining information from different sensors.

To solve the above mentioned problems, an adaptive variational Bayesian Student’s t-based cubature information filter (VBST-CIF) algorithm is developed. Furthermore a variational Bayesian Student’s t-based cubature information feedback fusion (VBST-CIFF) algorithm is proposed for nonlinear multi-sensor systems with heavy-tailed noise. Key contributions of this paper are the following.

(1) The information filter form is adopted for simplified computation and facilitation of multi-sensor fusion.
(2) A novel VBST-CIF algorithm for nonlinear systems with heavy-tailed noise is proposed. The SRC rule is introduced into the VB approach for joint estimation of states and noise statistics, by employing the ST distribution for modeling heavy-tailed noise.
(3) The proposed VBST-CIF algorithm is further extended to multi-sensor fusion, deriving a novel VBST-CIFF algorithm. The proposed VBST-CIFF algorithm facilitates multi-sensor fusion in nonlinear systems with different heavy-tailed measurement noise statistics for each sensor.
(4) Simulation and experimental results show that the proposed VBST-CIF/VBST-CIFF algorithms outperform conventional CIF and cubature information feedback fusion (CIFF) algorithms in scenarios concerning nonlinear systems with heavy-tailed noise.

The rest of the paper is organized as follows. First, the problem of state estimation for nonlinear systems with heavy-tailed measurement noise is formulated in Section 2. Then, the SRC rule and ST distribution are introduced in Section 3, wherein the ST distribution is utilized to model heavy-tailed noise and the VBST-CIF algorithm is also derived. Furthermore, a VBST-CIFF algorithm is provided for multi-sensor fusion of nonlinear systems with heavy-tailed noise in Section 4. In Section 5, the proposed VBST-CIF and VBST-CIFF algorithms are tested in nonlinear target tracking scenarios. Conclusions are provided in Section 6.

## 2. Problem Formulation

Consider the following model:

(1) $$\begin{array}{cc} x_{k} & =f\left(x_{k-1}\right)+w_{k-1} \end{array}$$

(2) $$\begin{array}{cc} y_{k} & =h\left(x_{k}\right)+v_{k} \end{array}$$

where: *k* denotes the discrete time index; $x_{k}$ and $y_{k}$ are the state and measurement vector, respectively; *f* and *h* denote state transition and, respectively, measurement function; $w_{k}$ is a zero-mean Gaussian process noise with covariance $Q_{k}$, i.e., $w_{k}∼N\left(0,Q_{k}\right)$, while $v_{k}$ is heavy-tailed measurement noise; N(μ,Σ) denotes a Gaussian random variable with mean μ and variance Σ. Moreover, it is assumed that $w_{i}$ and $v_{j}$ are uncorrelated for any *i* and *j*.

Conventional CIF can deal with state estimation in the Gaussian case. It has been introduced in [43] under the assumption that measurement noise has Gaussian distribution. Unfortunately, for systems (1) and (1) affected by heavy-tailed measurement noise, it may lead to non-accurate estimation because of the deviation between the assumed noise model and the actual one.

To this end, Huang et al. [38] have proposed an adaptive filter for linear systems. Nevertheless, such a filter is limited to linear single-sensor systems and is not suited to multi-sensor nonlinear systems. Although there are some methods for nonlinear state estimation, how to calculate the matrix parameter of heavy-tailed noise is still an open issue. Specifically, the posterior distribution parameters of the heavy-tailed noise contain a matrix related to the measurement matrix in traditional linear systems. Unfortunately, such a matrix cannot be directly obtained for nonlinear systems, thus implying that the analytical solution for joint estimation of system state as well as the noise parameters is not possible. On the other hand, for multi-sensor systems, multiple noise matrices need to be estimated, one for each sensor *s*. Hence, how to estimate these parameters together with the system state and fuse them still remains another open issue.

To conclude, the following problems still need to be addressed for the state estimation of a nonlinear system with heavy-tailed measurement noise.

(1) The conventional CIF assumes Gaussian measurement noise distribution. For systems with heavy-tailed noise, CIF is not able to estimate states and the noise statistics simultaneously.
(2) The current VB approach based on the conventional KF framework is not suited to nonlinear systems. In particular, the unknown noise matrix parameter is difficult to obtain in nonlinear systems;
(3) For multi-sensor systems with measurement noise signals of the various sensors possibly having different statistics, it is an open issue to estimate and fuse states as well as noise statistics.

Therefore, a novel adaptive filter for a multi-sensor nonlinear system with heavy-tailed measurement noise will be proposed in this paper. Results in this work will help to deal with state estimation and data fusion problems for nonlinear systems with heavy-tailed measurement noise.

## 3. Variational Bayesian Student’s t-Based Cubature Information Filter

### 3.1. Spherical-Radial Cubature Rule

The main ingredient of the CIF for nonlinear systems is the SRC rule. Based on the prior mean and covariance, an initial set of sampling points are selected and then propagated through the nonlinear function, thus providing transformed sampling points. Then, by weighting these transformed sampling points, the posterior mean and variance can be obtained. Specifically, the SRC rule operates as follows.

Nonlinear filtering involves integrals of this form:

(3) $$\begin{array}{cc} \mathrm{Int}(I) & =\int_{R^{n}}I\left(x\right)\;\exp\left(-x^{T}x\right)\;dx \end{array}$$

where I(·) is a nonlinear function and $R^{n}$ denotes the integration domain. Let x=rg with $g^{T}g=1$ and r∈[0,∞], so that $x^{T}x=r^{2}$, where *g* is a direction vector and *r* is the radius. Then, integration in (3) can be rewritten as:

(4) $$\begin{array}{cc} \mathrm{Int}(I) & =\int_{0}^{\infty }\int_{\Xi }I\left(rg\right)\;r^{n-1}\;\exp\left(-r^{2}\right)\;d\sigma \left(g\right)dr \end{array}$$

where $\Xi =\{g\in R^{n}|g^{T}g=1\}$ denotes an *n*-dimensional unit sphere surface and σ(·) is an element on Ξ. Thus, the integration in (4) can be transformed into a spherical integration:

(5) $$\begin{array}{cc} \mathrm{Sph}(r) & =\int_{\Xi }I\left(rg\right)\;d\sigma \left(g\right) \end{array}$$

and a radial integration:

(6) $$\begin{array}{cc} \mathrm{Int} & =\int_{0}^{\infty }\mathrm{Sph}\left(r\right)r^{n-1}\;\exp\left(-r^{2}\right)\;dr\;. \end{array}$$

Hence, for a Gaussian integration, it turns out that:

(7) $$\begin{array}{cc} \int_{R^{n}}I\left(x\right)\;N\left(x;\mu ,\Sigma \right)\;dx\; & ≅\frac{1}{2n}\sum_{i=1}^{2n}I\left(\sqrt{\Sigma }r_{i}+\mu \right) \end{array}$$

where: $\sqrt{\Sigma }$ can be obtained by performing Cholesky decomposition of Σ; $r_{i}=\sqrt{n}\;{\left\{e\right\}}_{i}$, ${\left\{e\right\}}_{i}$ being the *i*-th column of {e}, with, for example, $\left\{e\right\}=\left\{\left[\begin{array}{c} 1\\ 0 \end{array}\right],\left[\begin{array}{c} 0\\ 1 \end{array}\right],\left[\begin{array}{c} -1\\ 0 \end{array}\right],\left[\begin{array}{c} 0\\ -1 \end{array}\right]\right\}$ if $\left\{e\right\}\in R^{2}$.

### 3.2. Student’s t Distribution and Time Update

The ST distribution [38] can be described as:

(8) $$\begin{array}{cc} St(\alpha ;0,M,\kappa ) & =\int_{0}^{+\infty }N\left(\alpha ;0,M/\gamma \right)\;G\left(\gamma ;\frac{\kappa }{2},\frac{\kappa }{2}\right)\;d\gamma \end{array}$$

where: St(·;μ,M,κ) represents the ST PDF with mean μ, scale matrix *M*, and degree of freedom (DOF) parameter κ; γ denotes an auxiliary parameter; N(·;μ,M) is the Gaussian PDF with mean μ and variance *M*; G(·;a,b) denotes the Gamma PDF with parameters *a* and *b*.

Since the measurement noise has heavy-tailed characteristic, it can be modeled as an ST distribution with zero mean, scale matrix $R_{k}$, and DOF $\kappa_{k}$ [38] as parameters, i.e.,

(9) $$\begin{array}{cc} St(v_{k};0,R_{k},\kappa_{k}) & =\int_{0}^{+\infty }N\left(v_{k};0,R_{k}/\gamma_{k}\right)\;G\left(\gamma_{k};\frac{\kappa_{k}}{2},\frac{\kappa_{k}}{2}\right)d\gamma_{k} \end{array}$$

where $\gamma_{k}$ denotes the auxiliary parameter at time *k*.

Prior distributions of $R_{k}$, $\kappa_{k}$, as well as $\gamma_{k}$, are selected as inverse Wishart and, respectively, Gamma distribution, i.e.,

(10) $$\begin{array}{cc} p(R_{k-1}) & =IW(R_{k-1};{\hat{\delta }}_{k-1|k-1},{\hat{\Delta }}_{k-1|k-1}) \end{array}$$

(11) $$\begin{array}{cc} p(\gamma_{k-1}) & =G(\gamma_{k-1};{\hat{a}}_{k-1|k-1},{\hat{b}}_{k-1|k-1}) \end{array}$$

(12) $$\begin{array}{cc} p(\kappa_{k-1}) & =G(\kappa_{k-1};{\hat{\phi }}_{k-1|k-1},{\hat{\Phi }}_{k-1|k-1}) \end{array}$$

where IW(·) denotes the inverse Wishart distribution [44] and ${\hat{\delta }}_{k-1|k-1}$, ${\hat{\Delta }}_{k-1|k-1}$ are its parameters estimated at time k−1. Likewise, ${\hat{a}}_{k-1|k-1}$, ${\hat{b}}_{k-1|k-1}$ and ${\hat{\phi }}_{k-1|k-1}$, ${\hat{\Phi }}_{k-1|k-1}$ are the corresponding estimated distribution parameters of $\gamma_{k-1}$ and, respectively, $\kappa_{k-1}$.

Time updating is performed by applying a forgetting factor τ to the prior estimates [44], i.e.,

(13) $$\begin{array}{cc} {\hat{\delta }}_{k|k-1} & =\tau \left({\hat{\delta }}_{k-1|k-1}-n_{z}-1\right)+n_{z}+1 \end{array}$$

(14) $$\begin{array}{cc} {\hat{\Delta }}_{k|k-1} & =\tau {\hat{\Delta }}_{k-1|k-1} \end{array}$$

(15) $$\begin{array}{cc} {\hat{\phi }}_{k|k-1} & =\tau {\hat{\phi }}_{k-1|k-1} \end{array}$$

(16) $$\begin{array}{cc} {\hat{\Phi }}_{k|k-1} & =\tau {\hat{\Phi }}_{k-1|k-1} \end{array}$$

where: $n_{z}$ denotes the dimension of the measurement vector $y_{k}$; k|k−1 stands for prediction from time k−1 to *k*, while k−1|k−1 stands for the filtered estimation at k−1.

Suppose that the prior state estimate and SECM are ${\hat{x}}_{k-1|k-1}$ and $P_{k-1|k-1}$, respectively. By applying the SRC rule to the prior information, we have:

(17) $$\begin{array}{cc} P_{k-1|k-1} & =T_{k-1}T_{k-1}^{T} \end{array}$$

(18) $$\begin{array}{cc} \chi_{i,k-1} & =T_{k-1}r_{i}+{\hat{x}}_{k-1|k-1}\;\left(i=1,2,\cdots ,2n_{x}\right) \end{array}$$

(19) $$\begin{array}{cc} \Psi_{i,k-1} & =f(\chi_{i,k-1}) \end{array}$$

where: $T_{k-1}$ is a matrix obtained by performing the Cholesky decomposition of $P_{k-1|k-1}$; $r_{i}$ represents the *i*-th column of {e} in the SRC rule; $\chi_{i,k-1}$ denotes the *i*-th sampling point; $n_{x}$ is the dimension of $x_{k}$; $\Psi_{i,k-1}$ denotes the corresponding transformed *i*-th sampling obtained by propagating $\chi_{i,k-1}$ through f(·). To this end, the predicted ${\hat{x}}_{k|k-1}$ as well as $P_{k|k-1}$ are obtained as:

(20) $$\begin{array}{cc} {\hat{x}}_{k|k-1} & =\frac{1}{2n_{x}}\sum_{i=1}^{2n_{x}}\Psi_{i,k-1} \end{array}$$

(21) $$\begin{array}{cc} P_{k|k-1} & =\frac{1}{2n_{x}}\sum_{i=1}^{2n_{x}}\Psi_{i,k-1}\Psi_{i,k-1}^{T}-{\hat{x}}_{k|k-1}{\hat{x}}_{k|k-1}^{T}+Q_{k-1}\;. \end{array}$$

Thus, the predicted information matrix and state vector, $Z_{k|k-1}$ and $\zeta_{k|k-1}$, are obtained through

(22) $$\begin{array}{cc} Z_{k|k-1} & =P_{k|k-1}^{-1} \end{array}$$

(23) $$\begin{array}{cc} \zeta_{k|k-1} & =Z_{k|k-1}{\hat{x}}_{k|k-1}\;. \end{array}$$

### 3.3. Variational Bayesian Student’s t-Based Cubature Information Filter (VBST-CIF)

The joint posterior PDF of system state $x_{k}$, scale matrix $R_{k}$, DOF parameter $\kappa_{k}$, and  auxiliary parameter $\gamma_{k}$, denoted as $p(x_{k},R_{k},\kappa_{k},\gamma_{k}|y_{1:k})$, needs to be computed. However, for the nonlinear system (1) and (), an analytical solution for $p(x_{k},R_{k},\kappa_{k},\gamma_{k}|y_{1:k})$ is impossible to obtain. For the purpose of obtaining an approximate solution, the VB approach is introduced.

The key idea of the VB approach is to get an approximate posterior distribution q(·) via minimizing the Kullback–Leibler divergence (KLD) between the approximate q(·) and true one p(·) [36], i.e.,

(24) $$\begin{array}{cc} q^{*} & =\arg\;\min\;\mathrm{KLD}\;(q||p) \end{array}$$

where the KLD is defined as:

(25) $$\begin{array}{cc} \mathrm{KLD}\;(q||p) & \equiv \int q\left(\cdot \right)\;\log\;\frac{q(\cdot )}{p(\cdot )}\;d\left(\cdot \right)\;. \end{array}$$

Hence, the problem can be transformed into searching an approximate q(·) that minimizes the KLD between itself and the true p(·), i.e.,

(26) $$\begin{array}{cc} q^{*}\left(\cdot \right) & =\arg\;\min\;(q\left(\cdot \right)\;||\;p\left(x_{k},R_{k},\kappa_{k},\gamma_{k}|y_{1:k}\right))\;. \end{array}$$

The joint posterior distribution of the parameters to be estimated is approximated via the following factored form [36]:

(27) $$\begin{array}{c} p\left(x_{k},R_{k},\kappa_{k},\gamma_{k}|y_{1:k}\right)≅q_{x}\left(x_{k}\right)\;q_{R}\left(R_{k}\right)\;q_{\kappa }\left(\kappa_{k}\right)\;q_{\gamma }\left(\gamma_{k}\right) \end{array}$$

where $q_{x}\left(\cdot \right)$, $q_{R}\left(\cdot \right)$, $q_{\kappa }\left(\cdot \right)$, and $q_{\gamma }\left(\cdot \right)$ represent approximate posterior PDFs for $x_{k}$, $R_{k}$, $\kappa_{k}$, and $\gamma_{k}$, respectively. By applying logarithm to (27), it can be deduced that:

(28) $$\begin{array}{cc} \log\;q(\xi ) & =E_{\Omega^{\left(-\xi \right)}}\left(\log\;p\left(\Omega ,y_{1:k}\right)\right)+c_{\xi }\\ \Omega & \equiv \{x_{k},R_{k},\kappa_{k},\gamma_{k}\} \end{array}$$

where: Ω is the set of parameters $\left(x_{k},R_{k},\kappa_{k},\gamma_{k}\right)$ that need to be estimated; ξ is an element of Ω; $\Omega^{\left(-\xi \right)}$ denotes the complementary set of ξ in Ω; E(·) denotes the expectation operator; $c_{\xi }$ is a quantity that is independent of ξ.

By exploiting the hierarchical Gaussian form in [38], it can be obtained that:

(29) $$\begin{array}{cc} \; & p(x_{k},R_{k},\kappa_{k},\gamma_{k},y_{1:k})\\ \; & =N\left(y_{k};h\left(x_{k}\right),\frac{R_{k}}{\gamma_{k}}\right)\;N\left(x_{k};{\hat{x}}_{k|k-1},P_{k|k-1}\right)IW\left(R_{k};\delta_{k},\Delta_{k}\right)\;G\left(\gamma_{k};\frac{\kappa_{k}}{2},\frac{\kappa_{k}}{2}\right)G\left(\kappa_{k};\phi_{k},\Phi_{k})\;p(y_{1:k-1}\right) \end{array}$$

Setting $\xi =R_{k}$,

(30) $$\begin{array}{cc} \log\;q^{\left(j+1\right)}\left(\xi \right)= & \;\log\;q^{\left(j+1\right)}\left(R_{k}\right)\\ = & -0.5\;\left({\hat{\delta }}_{k|k-1}+n_{z}+2\right)\;\log\left(R_{k}\right)-0.5\;\mathrm{tr}\;\left\{\left[{\hat{\Delta }}_{k|k-1}+E^{\left(j+1\right)}\left(\gamma_{k}\right)\;B_{k}^{\left(j\right)}\right]\;R_{k}^{-1}\right\}+c_{R} \end{array}$$

from which we have:

(31) $$\begin{array}{cc} q^{\left(j+1\right)}\left(R_{k}\right) & =IW\left(R_{k};{\hat{\delta }}_{k|k}^{\left(j+1\right)},{\hat{\Delta }}_{k|k}^{\left(j+1\right)}\right) \end{array}$$

whose parameters ${\hat{\delta }}_{k|k}^{\left(j+1\right)}$ and ${\hat{\Delta }}_{k|k}^{\left(j+1\right)}$ can be obtained by:

(32) $$\begin{array}{cc} {\hat{\delta }}_{k|k}^{\left(j+1\right)} & ={\hat{\delta }}_{k|k-1}+1 \end{array}$$

(33) $$\begin{array}{cc} {\hat{\Delta }}_{k|k}^{\left(j+1\right)} & ={\hat{\Delta }}_{k|k-1}+E^{\left(j+1\right)}\left(\gamma_{k}\right)B_{k}^{\left(j\right)} \end{array}$$

where $B_{k}^{\left(j\right)}$ can be obtained via the SRC rule as follows:

(34) $$\begin{array}{cc} P_{k|k}^{\left(j\right)} & =T_{k}T_{k}^{T} \end{array}$$

(35) $$\begin{array}{cc} \chi_{i,k}^{\left(j\right)} & =T_{k}r_{i}+{\hat{x}}_{k|k}^{\left(j\right)} \end{array}$$

(36) $$\begin{array}{cc} Y_{i,k}^{\left(j\right)} & =h(\chi_{i,k}^{\left(j\right)}) \end{array}$$

(37) $$\begin{array}{cc} {\hat{y}}_{k|k}^{\left(j\right)} & =\frac{1}{2n_{x}}\sum_{i=1}^{2n_{x}}Y_{i,k}^{\left(j\right)} \end{array}$$

(38) $$\begin{array}{cc} B_{k}^{\left(j\right)} & =\frac{1}{2n_{x}}\sum_{i=1}^{2n_{x}}Y_{i,k}^{\left(j\right)}{Y_{i,k}^{\left(j\right)}}^{T}-{\hat{y}}_{k|k}^{\left(j\right)}{\left({\hat{y}}_{k|k}^{\left(j\right)}\right)}^{T}. \end{array}$$

Next, setting $\xi =\gamma_{k}$, we get:

(39) $$\begin{array}{cc} \log\;q^{\left(j+1\right)}\left(\xi \right)= & \;\log\;q^{\left(j+1\right)}\left(\gamma_{k}\right)\\ = & \;\left(\frac{n_{z}+E^{\left(j\right)}\left(\kappa_{k}\right)}{2}-1\right)\;\log\left(\gamma_{k}\right)-0.5\;\left\{\mathrm{tr}\;\left[B_{k}^{\left(j\right)}E^{\left(j\right)}\left(R_{k}^{-1}\right)\right]+E^{\left(j\right)}\left(\kappa_{k}\right)\right\}\gamma_{k}+c_{\gamma } \end{array}$$

from which:

(40) $$\begin{array}{cc} q^{\left(j+1\right)}\left(\gamma_{k}\right) & =G\left(\gamma_{k};{\hat{a}}_{k|k}^{\left(j+1\right)},{\hat{b}}_{k|k}^{\left(j+1\right)}\right) \end{array}$$

where:

(41) $$\begin{array}{cc} {\hat{a}}_{k|k}^{\left(j+1\right)} & =0.5\;(n_{z}+E^{\left(j\right)}\left(\kappa_{k}\right)) \end{array}$$

(42) $$\begin{array}{cc} {\hat{b}}_{k|k}^{\left(j+1\right)} & =-0.5\;\{\mathrm{tr}\;\left[B_{k}^{\left(j\right)}E^{\left(j\right)}\left(R_{k}^{-1}\right)\right]+E^{\left(j\right)}\left(\kappa_{k}\right)\}. \end{array}$$

Then, setting $\xi =\kappa_{k}$, we get

(43) $$\begin{array}{cc} \log\;q^{\left(j+1\right)}\left(\xi \right)= & \log\;q^{\left(j+1\right)}\left(\kappa_{k}\right)\\ = & \frac{\kappa_{k}}{2}\;\log\;\left(\frac{\kappa_{k}}{2}\right)-\log\;\Gamma \left(\frac{\kappa_{k}}{2}\right)\\ \; & +\left(\frac{\kappa_{k}}{2}-1\right)\;E^{\left(j+1\right)}\left(\log\;\left(\gamma_{k}\right)\right)-\frac{\kappa_{k}}{2}E^{\left(j+1\right)}\left(\gamma_{k}\right)+\left({\hat{\phi }}_{k|k-1}-1\right)\;\log\;\left(\kappa_{k}\right)-{\hat{\Phi }}_{k|k-1}\kappa_{k}+c_{\kappa } \end{array}$$

from which we have:

(44) $$\begin{array}{cc} q^{\left(j+1\right)}\left(\kappa_{k}\right) & =G\left(\kappa_{k};{\hat{\phi }}_{k|k}^{\left(j+1\right)},{\hat{\Phi }}_{k|k}^{\left(j+1\right)}\right) \end{array}$$

where:

(45) $$\begin{array}{cc} {\hat{\phi }}_{k|k}^{\left(j+1\right)} & ={\hat{\phi }}_{k|k-1}+0.5 \end{array}$$

(46) $$\begin{array}{cc} {\hat{\Phi }}_{k|k}^{\left(j+1\right)} & ={\hat{\Phi }}_{k|k-1}-0.5E^{\left(j+1\right)}\left(\log\left(\gamma_{k}\right)\right)+0.5E^{\left(j+1\right)}\left(\gamma_{k}\right)-0.5. \end{array}$$

Finally, setting $\xi =x_{k}$,

(47) $$\begin{array}{cc} \log\;q^{\left(j+1\right)}\left(\xi \right)= & \;\log\;q^{\left(j+1\right)}\left(x_{k}\right)\\ = & -0.5{\left(x_{k}-{\hat{x}}_{k|k-1}\right)}^{T}E^{\left(j\right)}\left(P_{k|k-1}^{-1}\right)\left(x_{k}-{\hat{x}}_{k|k-1}\right)\\ \; & -0.5\;E^{\left(j\right)}\left(\gamma_{k}\right){\left(y_{k}-h\left(x_{k}\right)\right)}^{T}E^{\left(j\right)}\left(R_{k}^{-1}\right)\left(y_{k}-h\left(x_{k}\right)\right)+c_{x} \end{array}$$

according to which we have:

(48) $$\begin{array}{c} q^{\left(j+1\right)}\left(x_{k}\right)=N\left(x_{k};{\hat{x}}_{k|k}^{\left(j+1\right)},P_{k|k}^{\left(j+1\right)}\right) \end{array}$$

where ${\hat{x}}_{k|k}^{\left(j+1\right)}$ and $P_{k|k}^{\left(j+1\right)}$ are the estimated state and SECM in the *j*-th VB iteration, respectively.

In order to obtain ${\hat{x}}_{k|k}^{\left(j+1\right)}$ and $P_{k|k}^{\left(j+1\right)}$, the associated information matrix and information vector contributions $I_{k}^{\left(j+1\right)}$ and, respectively, $\iota_{k}^{\left(j+1\right)}$ should be first computed. By utilizing a linearized error propagation method [26], they can be obtained as follows:

(49) $$\begin{array}{cc} I_{k}^{\left(j+1\right)} & =H_{k}^{T}{\left({\tilde{R}}_{k|k}^{\left(j+1\right)}\right)}^{-1}H_{k} \end{array}$$

(50) $$\begin{array}{cc} \iota_{k}^{\left(j+1\right)} & =H_{k}^{T}{\left({\tilde{R}}_{k|k}^{\left(j+1\right)}\right)}^{-1}\left(y_{k}-{\hat{y}}_{k|k-1}+H_{k}{\hat{x}}_{k|k-1}\right) \end{array}$$

where ${\tilde{R}}_{k|k}^{\left(j+1\right)}$ denotes the estimated scale matrix and $H_{k}$ a pseudo-measurement matrix. Then, exploiting (31) and a property of the inverse Wishart distribution, it can be obtained that:

(51) $$\begin{array}{cc} E^{\left(j+1\right)}\left(R_{k}^{-1}\right) & =\left({\hat{\delta }}_{k|k}^{\left(j+1\right)}-n_{z}-1\right){\left({\hat{\Delta }}_{k|k}^{\left(j+1\right)}\right)}^{-1}\;. \end{array}$$

Similarly, taking into account (40) and (44) as well as a property of the Gamma distribution, we have:

(52) $$\begin{array}{cc} E^{\left(j+1\right)}\left(\kappa_{k}\right) & ={\hat{\phi }}_{k|k}^{\left(j+1\right)}/{\hat{\Phi }}_{k|k}^{\left(j+1\right)} \end{array}$$

(53) $$\begin{array}{cc} E^{\left(j+1\right)}\left(\gamma_{k}\right) & ={\hat{a}}_{k|k}^{\left(j+1\right)}/{\hat{b}}_{k|k}^{\left(j+1\right)}\;. \end{array}$$

Then, ${\tilde{R}}_{k|k}^{\left(j+1\right)}$ is computed as:

(54) $$\begin{array}{cc} {\tilde{R}}_{k|k}^{\left(j+1\right)} & ={\left({\hat{\delta }}_{k|k}^{\left(j+1\right)}-n_{z}-1\right)}^{-1}{\hat{\Delta }}_{k|k}^{\left(j+1\right)}\;. \end{array}$$

Moreover, the pseudo-measurement matrix $H_{k}$ in (49) and (50) can be obtained as:

(55) $$\begin{array}{cc} H_{k} & =P_{xz,k|k-1}^{T}Z_{k|k-1} \end{array}$$

where $P_{xz,k|k-1}$ is computed by means of the SRC rule as follows:

(56) $$\begin{array}{cc} P_{k|k-1} & =T_{k|k-1}T_{k|k-1}^{T} \end{array}$$

(57) $$\begin{array}{cc} \chi_{i,k|k-1} & =T_{k|k-1}r_{i}+{\hat{x}}_{k|k-1} \end{array}$$

(58) $$\begin{array}{cc} Y_{i,k|k-1} & =h(\chi_{i,k|k-1}) \end{array}$$

(59) $$\begin{array}{cc} {\hat{y}}_{k|k-1} & =\frac{1}{2n_{x}}\sum_{i=1}^{2n_{x}}Y_{i,k|k-1} \end{array}$$

(60) $$\begin{array}{cc} P_{xz,k|k-1} & =\frac{1}{2n_{x}}\sum_{i=1}^{2n_{x}}\chi_{i,k-1}Y_{i,k-1}^{T}-{\hat{x}}_{k|k-1}{\hat{y}}_{k|k-1}^{T}\;. \end{array}$$

Then, the information matrix and vector are updated as follows:

(61) $$\begin{array}{cc} Z_{k|k}^{\left(j+1\right)} & =Z_{k|k-1}+I_{k}^{\left(j+1\right)} \end{array}$$

(62) $$\begin{array}{cc} \zeta_{k|k}^{\left(j+1\right)} & =\zeta_{k|k-1}+\iota_{k}^{\left(j+1\right)}\;. \end{array}$$

Finally, we have:

(63) $$\begin{array}{cc} {\hat{x}}_{k|k}^{\left(j+1\right)} & =Z_{k|k}^{\left(j+1\right)}\backslash \zeta_{k|k}^{\left(j+1\right)} \end{array}$$

(64) $$\begin{array}{cc} P_{k|k}^{\left(j+1\right)} & =Z_{k|k}^{\left(j+1\right)}\backslash I_{n_{x}} \end{array}$$

where $I_{n_{x}}$ denotes the $n_{x}\times n_{x}$ identity matrix. To summarize the above developments, the pseudocode of VBST-CIF is reported in Algorithm 1.

**Algorithm 1** Time-recursion of VBST-CIF**Input:** ${\hat{x}}_{k-1|k-1}$, $P_{k-1|k-1}$, ${\hat{\delta }}_{k-1|k-1}$, ${\hat{\Delta }}_{k-1|k-1}$, ${\hat{\phi }}_{k-1|k-1}$, ${\hat{\Phi }}_{k-1|k-1}$, $y_{k}$, $Q_{k-1}$, τ **Step 1: Time update:** **(1)** Compute ${\hat{x}}_{k|k-1}$ and $P_{k|k-1}$ based on the SRC rule by (17)–(21). **(2)** Compute $Z_{k|k-1}$ and $\zeta_{k|k-1}$ by (22) and (23). **(3)** Compute ${\hat{\delta }}_{k|k-1}$ and ${\hat{\Delta }}_{k|k-1}$ by (13) and (14). **(4)** Compute ${\hat{\phi }}_{k|k-1}$ and ${\hat{\Phi }}_{k|k-1}$ by (15) and (16). **Step 2: Variational fixed point iterations:** **Initialize:**  ${\hat{x}}_{k|k}^{\left(0\right)}={\hat{x}}_{k|k-1}$, $P_{k|k}^{\left(0\right)}=P_{k|k-1}$, ${\hat{\delta }}_{k|k}^{\left(0\right)}={\hat{\delta }}_{k|k-1}$, ${\hat{\Delta }}_{k|k}^{\left(0\right)}={\hat{\Delta }}_{k|k-1}$, ${\hat{\phi }}_{k|k}^{\left(0\right)}={\hat{\phi }}_{k|k-1}$, ${\hat{\Phi }}_{k|k}^{\left(0\right)}={\hat{\Phi }}_{k|k-1}$ **Measurement update:** **(1)** Perform Cholesky decomposition of $P_{k|k-1}$ and cubature sampling via (56) and (57). **(2)** Compute $P_{xz,k|k-1}$ by (58)–(60). **(3)** Variational parameter update **for**
j=0,1,⋯,N−1
**do**  **(a)** Compute $q^{\left(j+1\right)}\left(R_{k}\right)$ by (31):    Compute $B_{k}^{\left(j\right)}$ by (34)–(38).    Update ${\hat{\delta }}_{k|k}^{\left(j+1\right)}$ and ${\hat{\Delta }}_{k|k}^{\left(j+1\right)}$ by (33) and (33).  **(b)** Compute $q^{\left(j+1\right)}\left(\gamma_{k}\right)$ by (40):    Compute ${\hat{a}}_{k|k}^{\left(j+1\right)}$ and ${\hat{b}}_{k|k}^{\left(j+1\right)}$ by (41) and (42).  **(c)** Compute $q^{\left(j+1\right)}\left(\kappa_{k}\right)$ by (44):    Update ${\hat{\phi }}_{k|k}^{\left(j+1\right)}$ and ${\hat{\Phi }}_{k|k}^{\left(j+1\right)}$ by (45) and (46).  **(d)** Compute $q^{\left(j+1\right)}\left(x_{k}\right)$ by (48):  Compute ${\tilde{R}}_{k|k}^{\left(j+1\right)}$ by (54).    Compute $H_{k}$ by (55).   Update $Z_{k|k}^{\left(j+1\right)}$ and $\zeta_{k|k}^{\left(j+1\right)}$ by (61) and (61).    Compute ${\hat{x}}_{k|k}^{\left(j+1\right)}$ and $P_{k|k}^{\left(j+1\right)}$ by (63) and (64). **end for:** **Step 3: State update:**  ${\hat{x}}_{k|k}={\hat{x}}_{k|k}^{\left(N\right)}$, $P_{k|k}=P_{k|k}^{\left(N\right)}$, ${\hat{\delta }}_{k|k}={\hat{\delta }}_{k|k}^{\left(N\right)}$, ${\hat{\Delta }}_{k|k}={\hat{\Delta }}_{k|k}^{\left(N\right)}$, ${\hat{\phi }}_{k|k}={\hat{\phi }}_{k|k}^{\left(N\right)}$, ${\hat{\Phi }}_{k|k}={\hat{\Phi }}_{k|k}^{\left(N\right)}$**Output:** ${\hat{x}}_{k|k}$, $P_{k|k}$, ${\hat{\delta }}_{k|k}$, ${\hat{\Delta }}_{k|k}$, ${\hat{\phi }}_{k|k}$, ${\hat{\Phi }}_{k|k}$

## 4. Variational Bayesian Student’s t-Based Cubature Information Feedback Fusion (VBST-CIFF)

### 4.1. Multi-Sensor Cubature Information Feedback Fusion (CIFF)

**Figure 1:**
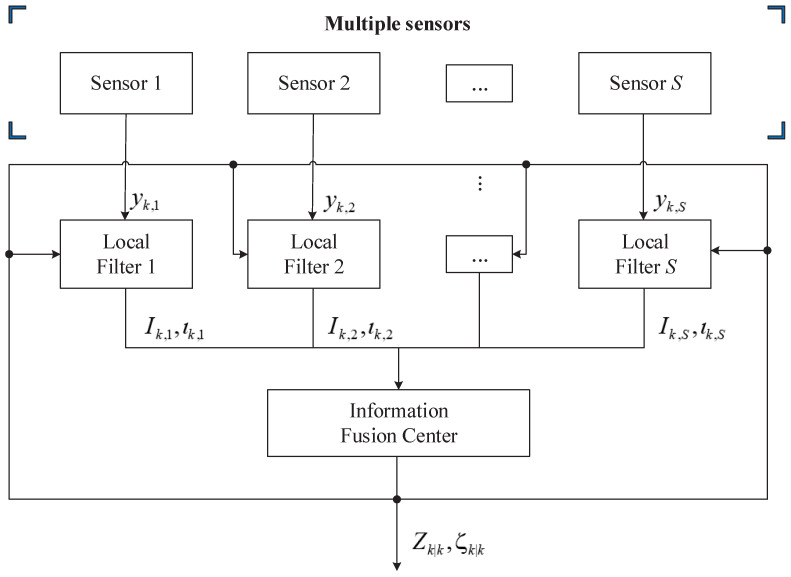
Multi-sensor information feedback fusion.

The information feedback fusion (IFF) algorithm derived in this section is suitable for multi-sensor fusion. The overall structure of the IFF is schematized in Figure 1, where each local filter receives raw measurements from the relative sensor and produces sensor-dependent variables to be used by the information fusion center in order to update the information matrix and state vector, which are then fed back to the local filters.

Based on the structure of IFF, the CIFF algorithm is derived as follows. Consider that there are *S* sensors. For each sensor *s*, s∈{1,2,⋯,S}, a measurement $y_{k,s}$ is available at time *k*, i.e.,

(65) $$\begin{array}{cc} y_{k,s} & =h_{s}\left(x_{k}\right)+v_{k,s} \end{array}$$

where $h_{s}\left(\cdot \right)$ denotes the measurement function of sensor *s*, and  $v_{k,s}$ the corresponding measurement noise. It is assumed that $v_{k,m}$ and $v_{k,n}$ are uncorrelated for any m≠n. Let, at time k−1, the state estimate and SECM for all sensors be ${\hat{x}}_{k-1|k-1}$ and, respectively, $P_{k-1|k-1}$. Correspondingly, the initial information vector and matrix for all sensors are $\zeta_{k-1|k-1}$ and $Z_{k-1|k-1}$, respectively. Then, by exploiting the SRC rule, predicted $\zeta_{k-1|k}$ and $Z_{k-1|k}$ can be obtained, i.e.,

(66) $$\begin{array}{cc} {\left(Z_{k-1|k-1}\right)}^{-1} & =T_{k-1}T_{k-1}^{T} \end{array}$$

where $Z_{k-1|k-1}$ is the inverse of $P_{k-1|k-1}$. Transforming $\chi_{i,k-1}$ in (18) through f(·), we have $\Psi_{i,k-1}$ in (19). Then, predicted $Z_{k|k-1}$ and $\zeta_{k|k-1}$, are computed via (22) and (23).

At the fusion center, the estimated information vector and matrix can, therefore, be updated as follows:

(67) $$\begin{array}{cc} \zeta_{k|k} & =\zeta_{k|k-1}+\sum_{s=1}^{S}\iota_{k,s} \end{array}$$

(68) $$\begin{array}{cc} Z_{k|k} & =Z_{k|k-1}+\sum_{s=1}^{S}I_{k,s} \end{array}$$

where: the local sensor contributions $I_{k,s}$ and $\iota_{k,s}$ are given by:

(69) $$\begin{array}{cc} I_{k,s} & =H_{k,s}^{T}R_{k,s}^{-1}H_{k} \end{array}$$

(70) $$\begin{array}{cc} \iota_{k,s} & =H_{k,s}^{T}R_{k,s}^{-1}\left(y_{k,s}-{\hat{y}}_{k|k-1,s}+H_{k}{\hat{x}}_{k|k-1}\right)\;; \end{array}$$

the pseudo-measurement matrix $H_{k,s}$ of sensor *s* can be obtained through the statistical linearized error propagation approach as in Section 3.3; $R_{k,s}$ denotes the MNCM of sensor *s*; ${\hat{y}}_{k|k-1,s}$ can be obtained by the SRC rule as in the conventional CIF [25].

### 4.2. Variational Bayesian Student’s t-Based Cubature Information Feedback Fusion (VBST-CIFF)

Consider now that each sensor *s* is characterized by heavy-tailed measurement noise $v_{k,s}$. In the time-update, the parameters ${\hat{\delta }}_{k|k-1,s}$, ${\hat{\Delta }}_{k|k-1,s}$, ${\hat{\phi }}_{k|k-1,s}$, and ${\hat{\Phi }}_{k|k-1,s}$ for sensor *s*, can be obtained via (71)–(74), respectively, i.e.,

(71) $$\begin{array}{cc} {\hat{\delta }}_{k|k-1,s} & =\tau \left({\hat{\delta }}_{k-1|k-1,s}-n_{z}-1\right)+n_{z}+1 \end{array}$$

(72) $$\begin{array}{cc} {\hat{\Delta }}_{k|k-1,s} & =\tau {\hat{\Delta }}_{k-1|k-1,s} \end{array}$$

(73) $$\begin{array}{cc} {\hat{\phi }}_{k|k-1,s} & =\tau {\hat{\phi }}_{k-1|k-1,s} \end{array}$$

(74) $$\begin{array}{cc} {\hat{\Phi }}_{k|k-1,s} & =\tau {\hat{\Phi }}_{k-1|k-1,s}\;. \end{array}$$

Then, in the measurement update, the estimated scale matrix for sensor *s* is given by:

(75) $$\begin{array}{cc} {\tilde{R}}_{k|k,s}^{\left(j+1\right)} & ={\left({\hat{\delta }}_{k|k,s}^{\left(j+1\right)}-n_{z}-1\right)}^{-1}{\hat{\Delta }}_{k|k,s}^{\left(j+1\right)} \end{array}$$

where ${\hat{\delta }}_{k|k,s}^{\left(j+1\right)}$ and ${\hat{\Delta }}_{k|k,s}^{\left(j+1\right)}$ can be obtained via:

(76) $$\begin{array}{cc} {\hat{\delta }}_{k|k,s}^{\left(j+1\right)} & ={\hat{\delta }}_{k|k-1}+1 \end{array}$$

(77) $$\begin{array}{cc} {\hat{\Delta }}_{k|k,s}^{\left(j+1\right)} & ={\hat{\Delta }}_{k|k-1}+E^{\left(j+1\right)}\left(\gamma_{k,s}\right)B_{k,s}^{\left(j\right)} \end{array}$$

where $B_{k,s}^{\left(j\right)}$ can be derived in the same way as in (34)–(38) with cubature sampling points transformed through $h_{s}\left(\cdot \right)$ as:

(78) $$\begin{array}{cc} Y_{i,k|k-1,s} & =h_{s}\left(\chi_{i,k|k-1,s}\right) \end{array}$$

(79) $$\begin{array}{cc} {\hat{y}}_{k|k-1,s} & =\frac{1}{2n_{z}}\sum_{i=1}^{2n_{z}}Y_{i,k|k-1,s} \end{array}$$

in which the original sampling points $\chi_{i,k|k-1,s}$ are given by (56) and (57). Furthermore, $E^{\left(j+1\right)}\left(\gamma_{k,s}\right)$ for sensor *s* can be obtained by:

(80) $$\begin{array}{cc} E^{\left(j+1\right)}\left(\gamma_{k,s}\right) & ={\hat{a}}_{k|k,s}^{\left(j+1\right)}/{\hat{b}}_{k|k,s}^{\left(j+1\right)} \end{array}$$

where,

(81) $$\begin{array}{cc} {\hat{a}}_{k|k,s}^{\left(j+1\right)} & =0.5\;(n_{z}+E^{\left(j\right)}\left(\kappa_{k,s}\right)) \end{array}$$

(82) $$\begin{array}{cc} {\hat{b}}_{k|k,s}^{\left(j+1\right)} & =-0.5\;\{\mathrm{tr}\;\left[B_{k,s}^{\left(j+1\right)}E^{\left(j\right)}\left(R_{k,s}^{-1}\right)\right]+E^{\left(j\right)}\left(\kappa_{k,s}\right)\} \end{array}$$

and $E^{\left(j+1\right)}\left(\kappa_{k,s}\right)$ is given by:

(83) $$\begin{array}{cc} E^{\left(j+1\right)}\left(\kappa_{k,s}\right) & ={\hat{\phi }}_{k|k,s}^{\left(j+1\right)}/{\hat{\Phi }}_{k|k,s}^{\left(j+1\right)} \end{array}$$

in which:

(84) $$\begin{array}{cc} {\hat{\phi }}_{k|k,s}^{\left(j+1\right)} & ={\hat{\phi }}_{k|k-1,s}+0.5 \end{array}$$

(85) $$\begin{array}{cc} {\hat{\Phi }}_{k|k,s}^{\left(j+1\right)} & ={\hat{\Phi }}_{k|k-1,s}-0.5E^{\left(j+1\right)}\left(\log\left(\gamma_{k,s}\right)\right)+0.5E^{\left(j+1\right)}\left(\gamma_{k,s}\right)-0.5\;. \end{array}$$

The pseudo-measurement matrix for sensor *s* turns out to be:

(86) $$\begin{array}{cc} H_{k,s} & =P_{xz,k|k-1,s}^{T}Z_{k|k-1} \end{array}$$

where $P_{xz,k|k-1,s}^{T}$ can be obtained via

(87) $$\begin{array}{cc} P_{xz,k|k-1,s} & =\frac{1}{2n_{x}}\sum_{i=1}^{2n_{x}}\chi_{i,k-1}Y_{i,k-1,s}^{T}-{\hat{x}}_{k|k-1}{\hat{y}}_{k|k-1,s}^{T}\;. \end{array}$$

Hence, the information matrix and vector contributions due to sensor *s* can be derived as follows:

(88) $$\begin{array}{cc} I_{k,s}^{\left(j+1\right)} & =H_{k,s}^{T}{\left({\tilde{R}}_{k|k,s}^{\left(j+1\right)}\right)}^{-1}H_{k,s} \end{array}$$

(89) $$\begin{array}{cc} \iota_{k,s}^{\left(j+1\right)} & =H_{k,s}^{T}{\left({\tilde{R}}_{k|k,s}^{\left(j+1\right)}\right)}^{-1}\left(y_{k,s}-{\hat{y}}_{k|k-1,s}+H_{k,s}{\hat{x}}_{k|k-1}\right) \end{array}$$

from which the information matrix and vector can be updated according to

(90) $$\begin{array}{cc} Z_{k|k}^{\left(j+1\right)} & =Z_{k|k-1}+\sum_{s=1}^{S}I_{k,s}^{\left(j+1\right)} \end{array}$$

(91) $$\begin{array}{cc} \zeta_{k|k}^{\left(j+1\right)} & =\zeta_{k|k-1}+\sum_{s=1}^{S}\iota_{k,s}^{\left(j+1\right)}\;. \end{array}$$

**Figure 2:**
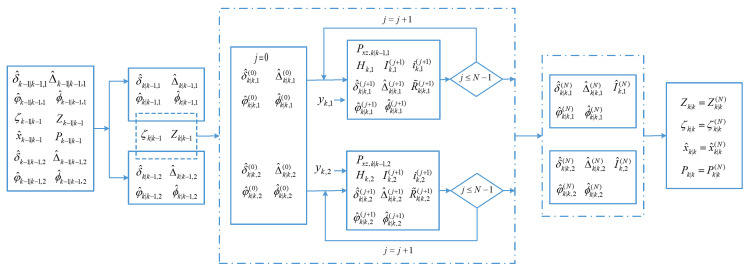
Flow-chart of proposed VBST-CIFF (variational Bayesian Student’s t-based-cubature information feedback fusion).

Finally, the global state estimate and covariance can be obtained by (63) and (64). To summarize the above developments, the flow-chart of the proposed VBST-CIFF is shown in Figure 2. Accordingly, the pseudocode is reported in Algorithm 2.

Please notice that VBST-CIFF is actually tightly-coupled (centralized) in that the raw measurements from all sensors are integrated into a single nonlinear information filter. Thanks to the adoption of the information filter form and the assumption that measurement noise signals of the various sensors are uncorrelated, the filter’s multi-sensor measurement update turns out to be decoupled into single-sensor updates as in (67) and (68). However, the overall filter is coupled through the information feedback of $Z_{k},\zeta_{k}$ into the local filters (see Figure 1).
**Algorithm 2** Time-recursion of VBST-CIFF**Input:** ${\hat{x}}_{k-1|k-1}$, $P_{k-1|k-1}$, $y_{k,s}$, τ, ${\hat{\delta }}_{k-1|k-1,s}$, ${\hat{\Delta }}_{k-1|k-1,s}$, ${\hat{\phi }}_{k-1|k-1,s}$, ${\hat{\Phi }}_{k-1|k-1,s}$, $Q_{k-1}$ **Step 1: Time update:** **(1)** Compute $Z_{k|k-1}$ and $\zeta_{k|k-1}$ by (22) and (23). **(2)** Compute ${\hat{\delta }}_{k|k-1,s}$ and ${\hat{\Delta }}_{k|k-1,s}$ by (71) and (72). **(3)** Compute ${\hat{\phi }}_{k|k-1,s}$ and ${\hat{\Phi }}_{k|k-1,s}$ by (73) and (74). **Step 2: Variational fixed-point iterations:** **Initialize:**  ${\hat{x}}_{k|k}^{\left(0\right)}={\hat{x}}_{k|k-1}$, $P_{k|k}^{\left(0\right)}=P_{k|k-1}$, ${\hat{\delta }}_{k|k,s}^{\left(0\right)}={\hat{\delta }}_{k|k-1,s}$, ${\hat{\Delta }}_{k|k,s}^{\left(0\right)}={\hat{\Delta }}_{k|k-1,s}$, ${\hat{\phi }}_{k|k,s}^{\left(0\right)}={\hat{\phi }}_{k|k-1,s}$, ${\hat{\Phi }}_{k|k,s}^{\left(0\right)}={\hat{\Phi }}_{k|k-1,s}$ **Measurement update:** **(1)** Perform Cholesky decomposition of $P_{k|k-1}$ and cubature sampling via (56) and (57). **(2)** Compute $P_{xz,k|k-1,s}$ by (87). **(3)** Variational parameter update: **for**
j=0,1,⋯,N−1
**do**   **(a)** Update $E^{\left(j\right)}\left(\kappa_{k,s}\right)$, ${\hat{\phi }}_{k|k,s}^{\left(j+1\right)}$, and ${\hat{\Phi }}_{k|k,s}^{\left(j+1\right)}$ by (83)–(85).   **(b)** Update $E^{\left(j+1\right)}\left(\gamma_{k,s}\right)$, ${\hat{a}}_{k|k,s}^{\left(j+1\right)}$, and ${\hat{b}}_{k|k,s}^{\left(j+1\right)}$ by (80)–(82).   **(c)** Update ${\tilde{R}}_{k|k,s}^{\left(j+1\right)}$, ${\hat{\delta }}_{k|k,s}^{\left(j+1\right)}$ and ${\hat{\Delta }}_{k|k,s}^{\left(j+1\right)}$ by (75)–(77).   **(d)** Update $H_{k,s}$ by (86).   **(e)** Update $I_{k,s}^{\left(j+1\right)}$ and $\iota_{k,s}^{\left(j+1\right)}$ by (88) and (89).   **(f)** Set variational parameters:     ${\hat{\delta }}_{k|k,s}={\hat{\delta }}_{k|k,s}^{\left(N\right)}$, ${\hat{\Delta }}_{k|k,s}={\hat{\Delta }}_{k|k,s}^{\left(N\right)}$, ${\hat{\phi }}_{k|k,s}={\hat{\phi }}_{k|k,s}^{\left(N\right)}$, ${\hat{\Phi }}_{k|k,s}={\hat{\Phi }}_{k|k,s}^{\left(N\right)}$ **end for**
 **Step 3: Fusion (at the global center):** **(1)** Update $Z_{k|k}^{\left(N\right)}$ and $\zeta_{k|k}^{\left(N\right)}$ by (90) and (91); **(2)** Update ${\hat{x}}_{k|k}^{\left(N\right)}$ and $P_{k|k}^{\left(N\right)}$ via (63) and (64). **(3)** Set:  ${\hat{x}}_{k|k}={\hat{x}}_{k|k}^{\left(N\right)}$, $P_{k|k}=P_{k|k}^{\left(N\right)}$, ${\hat{\delta }}_{k|k,s}={\hat{\delta }}_{k|k,s}^{\left(N\right)}$, ${\hat{\Delta }}_{k|k,s}={\hat{\Delta }}_{k|k,s}^{\left(N\right)}$, ${\hat{\phi }}_{k|k}={\hat{\phi }}_{k|k,s}^{\left(N\right)}$, ${\hat{\Phi }}_{k|k}={\hat{\Phi }}_{k|k,s}^{\left(N\right)}$**Output:**${\hat{x}}_{k|k}$, $P_{k|k}$, ${\hat{\delta }}_{k|k,s}$, ${\hat{\Delta }}_{k|k,s}$, ${\hat{\phi }}_{k|k,s}$, ${\hat{\Phi }}_{k|k,s}$

## 5. Simulation Results

### 5.1. VBST-CIF Single-Sensor Target Tracking

Consider a target car tracking scenario with just a single sensor (a 2D radar). The state of the car is taken as $x={\left[\varsigma ,\dot{\varsigma },\mu ,\dot{\mu },\omega \right]}^{T}$, where ς and μ are the Cartesian coordinates of position, while $\dot{\varsigma }$ and $\dot{\mu }$ represent the corresponding velocity, and ω is the turning rate. The car moves according to the coordinated turn (CT) model:

(92) $$\begin{array}{cc} x_{k} & =F\left(\omega_{k-1}\right)x_{k-1}+w_{k-1} \end{array}$$

with a turning-rate dependent state-transition matrix:

$$F\left(\omega \right)=\left[\begin{array}{ccccc} 1 & \frac{\sin(\omega )}{\omega } & 0 & -\frac{1-\cos(\omega )}{\omega } & 0\\ 0 & \cos(\omega ) & 0 & -\sin(\omega ) & 0\\ 0 & \frac{1-\cos(\omega )}{\omega } & 1 & \frac{\sin(\omega )}{\omega } & 0\\ 0 & \sin(\omega ) & 0 & \cos(\omega ) & 0\\ 0 & 0 & 0 & 0 & 1 \end{array}\right]$$

fixed at turning rate $\omega =10^{\circ }$; $w_{k-1}=N\left(0,Q_{k-1}\right)$;

$$Q_{k-1}=\left[\begin{array}{ccccc} \frac{p_{1}T^{3}}{3} & \frac{p_{1}T^{2}}{2} & 0 & 0 & 0\\ \frac{p_{1}T^{2}}{2} & T & 0 & 0 & 0\\ 0 & 0 & \frac{p_{1}T^{3}}{3} & \frac{p_{1}T^{2}}{2} & 0\\ 0 & 0 & \frac{p_{1}T^{2}}{2} & T & 0\\ 0 & 0 & 0 & 0 & p_{2}T \end{array}\right],$$

with noise intensity factors $p_{1}=0.1$, $p_{2}=1.75\times 10^{-4}$ and sampling interval T=1[s]. Conversely, the measurement function of the radar is:

(93) $$\begin{array}{cc} y_{k} & =\left[\begin{array}{c} \sqrt{\varsigma_{k}^{2}+\mu_{k}^{2}}\\ \mathrm{atan}2\left(\varsigma_{k},\mu_{k}\right) \end{array}\right]+v_{k} \end{array}$$

where: atan2(ς,μ) denotes the 4-quadrant inverse-tangent defined as the argument of the complex number ς+jμ, *j* denoting the imaginary unit; $v_{k}$ is heavy-tailed measurement noise such that:

(94) $$\begin{array}{c} v_{k}∼\left\{\begin{array}{cc} N\left(0,\Lambda_{k}\right), & \mathrm{with}\;\mathrm{probability}\;0.9\\ N\left(0,100\Lambda_{k}\right), & \mathrm{with}\;\mathrm{probability}\;0.1 \end{array}\right. \end{array}$$

with $\Lambda_{k}=\left[\begin{array}{cc} 4\;[m^{2}] & 0\\ 0 & 10^{-4}\;\left[{\mathrm{rad}}^{2}\right] \end{array}\right].$ Moreover, the initial values for state and SECM of the car are set as follows:

(95) $$\begin{array}{cc} x_{0} & ={\left[100\;\left[m\right],3\;\left[m/s\right],100\;\left[m\right],2\;\left[m/s\right],\frac{-10\pi }{180}\;\left[\mathrm{rad}\right]\;\right]}^{T} \end{array}$$

(96) $$\begin{array}{cc} P_{0} & =\left[\begin{array}{ccccc} 10\;[m^{2}] & 0 & 0 & 0 & 0\\ 0 & 1\;[m^{2}/s^{2}] & 0 & 0 & 0\\ 0 & 0 & 10\;[m^{2}] & 0 & 0\\ 0 & 0 & 0 & 1\;[m^{2}/s^{2}] & 0\\ 0 & 0 & 0 & 0 & 10^{-4}\;\left[{\mathrm{rad}}^{2}\right] \end{array}\right]. \end{array}$$

A total of 100 independent Monte Carlo trials have been carried out, with each trial lasting for t=50[s]. Furthermore, the root-mean-square-error (RMSE) is adopted to assess tracking precision. For variable *b* at time *k*, it is defined as:

(97) $$\begin{array}{cc} {\mathrm{RMSE}}_{b}\left(k\right) & =\sqrt{\frac{1}{N_{MC}}\sum_{i=1}^{N_{MC}}{\left(b_{k}^{i}-{\hat{b}}_{k}^{i}\right)}^{T}\left(b_{k}^{i}-{\hat{b}}_{k}^{i}\right)} \end{array}$$

where: $N_{MC}$ denotes the number of Monte Carlo trials; *b* denotes either the position $p={\left[\varsigma ,\mu \right]}^{T}$ or velocity $v={\left[\dot{\varsigma },\dot{\mu }\right]}^{T}$; and $b_{k}^{i}$ is the true value in the *i*-th trial at time *k*. Conversely, ${\hat{b}}_{k}^{i}$ represents the estimated value in the *i*-th trial at time *k*. Furthermore, the mean-root-mean-square-error (MRMSE) is used to measure the average RMSE during the whole simulation time. Specifically, MRMSE for variable *b* is defined as:

(98) $$\begin{array}{cc} {\mathrm{RMSE}}_{b} & =\frac{1}{t/T}\sum_{k=1}^{t/T}{\mathrm{RMSE}}_{b}\left(k\right). \end{array}$$

Simulation results are presented to compare the conventional CIF [43] with the proposed VBST-CIF. Simulation parameters have been chosen in the same way as in [33]. Specifically, in CIF, the process noise covariance *Q* and MNCM *R* are fixed to $Q=Q_{k-1}$ and $R=\Lambda_{k}$. Conversely, in the proposed VBST-CIF, $Q=Q_{k-1}$, while the scale matrix as well as other noise statistical parameters are adaptively estimated. Other parameters are set in the same way as in [33]: forgetting the factor τ=0.9; number of VB iterations N=10; initial noise parameters of the scale matrix $\delta =n_{z}+2=4$ and $\Delta =\Lambda_{k}$; initial noise parameters of the DOF parameter ϕ=5 and Φ=1.

**Figure 3:**
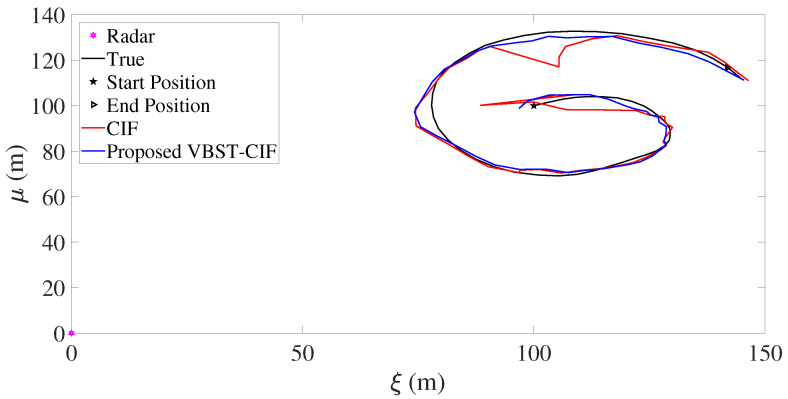
Target trajectory.

**Figure 4:**
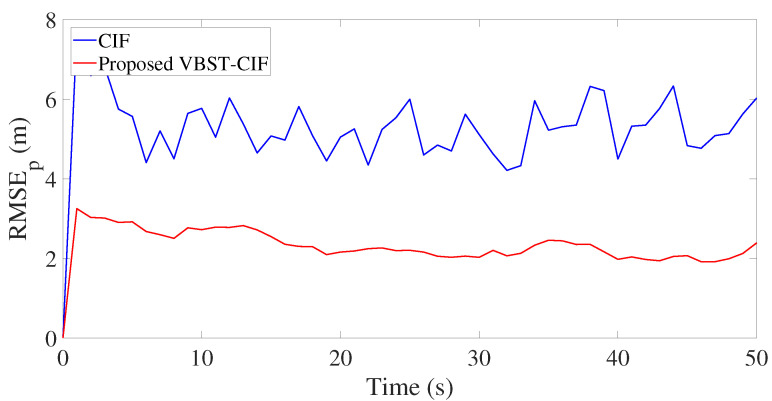
Position root-mean-square-error (RMSE) in single-sensor target tracking.

**Figure 5:**
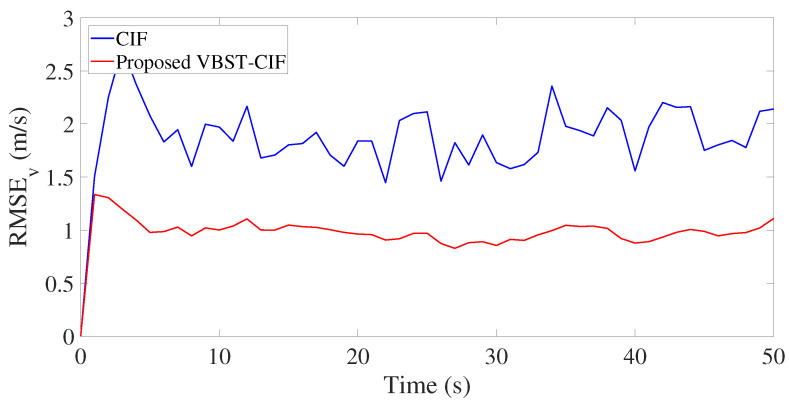
Velocity RMSE in single-sensor target tracking.

**Figure 6:**
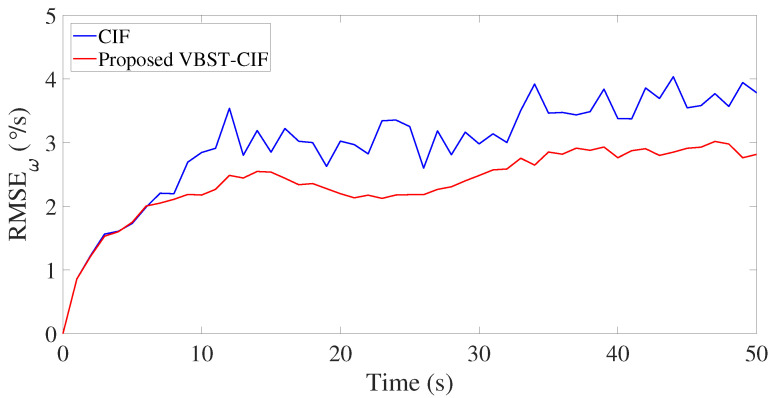
Turning-rate RMSE in single-sensor target tracking.

Results are presented in Figure 3, Figure 4, Figure 5 and Figure 6 and Table 1. Figure 3, Figure 4, Figure 5 and Figure 6 demonstrate that the proposed VBST-CIF outperforms conventional CIF in position, velocity, and turning-rate estimation. Precisely, Table 1 shows that VBST-CIF yields an MRMSE improvement with respect to conventional CIF of respectively 56% in position, 48% in velocity, and 20% in turning-rate.

### 5.2. VBST-CIFF Multi-Sensor Target Tracking

Let us now consider a multi-sensor target (car) tracking scenario with two (radar) sensors $S_{1}$ and $S_{2}$, located at (10,10) and respectively at the origin (0,0). The car’s state is taken as $x={\left[\varsigma ,\dot{\varsigma },\mu ,\dot{\mu }\right]}^{T}$ where ς, $\dot{\varsigma }$, μ, and $\dot{\mu }$ have the same same meaning as in Section 5.1. The state transition matrix in (92) is:

$$F=\left[\begin{array}{cccc} 1 & 0 & T & 0\\ 0 & 1 & 0 & T\\ 0 & 0 & 1 & 0\\ 0 & 0 & 0 & 1 \end{array}\right]\;.$$

The process noise covariance is $Q_{k-1}=I_{4}$, where $I_{4}$ denotes the 4×4 identity matrix. Furthermore, the measurement model is:

(99) $$\begin{array}{cc} y_{k,s} & =\left[\begin{array}{c} \sqrt{{\left(\varsigma_{k}-\varsigma^{s}\right)}^{2}+{\left(\mu_{k}-\mu^{s}\right)}^{2}}\\ atan2\left(\varsigma_{k},\mu_{k}\right) \end{array}\right]+v_{k,s} \end{array}$$

where: s=1,2 refers to $S_{1}$, $S_{2}$; $y_{k,s}$ denotes the measurement of radar *s* at time *k*; ${\left[\varsigma^{s},\mu^{s}\right]}^{T}$ refers to the known position of radar *s*, specifically ${\left[\varsigma^{1},\mu^{1}\right]}^{T}={\left[10,10\right]}^{T}$ and ${\left[\varsigma^{2},\mu^{2}\right]}^{T}={\left[0,0\right]}^{T}$; $v_{k,s}$ is the heavy-tailed noise of radar *s* at time *k* generated as follows:

(100) $$\begin{array}{c} v_{k,1}∼\left\{\begin{array}{cc} N\left(0,\Lambda_{k,1}\right), & \mathrm{with}\;\mathrm{probability}\;0.9\\ N\left(0,100\Lambda_{k,1}\right), & \mathrm{with}\;\mathrm{probability}\;0.1 \end{array}\right. \end{array}$$

(101) $$\begin{array}{c} v_{k,2}∼\left\{\begin{array}{cc} N\left(0,\Lambda_{k,2}\right), & \mathrm{with}\;\mathrm{probability}\;0.9\\ N\left(0,100\Lambda_{k,2}\right), & \mathrm{with}\;\mathrm{probability}\;0.1 \end{array}\right. \end{array}$$

where $\Lambda_{k,1}=\left[\begin{array}{cc} 15^{2}\;\left[m^{2}\right] & 0\\ 0 & 0.015^{2}\;\left[{\mathrm{rad}}^{2}\right] \end{array}\right]$ and $\Lambda_{k,2}=\left[\begin{array}{cc} 10^{2}\;\left[m^{2}\right] & 0\\ 0 & 0.01^{2}\;\left[{\mathrm{rad}}^{2}\right] \end{array}\right].$ Initial values for $x_{0}$ and $P_{0}$ are set as follows:

(102) $$\begin{array}{cc} x_{0} & ={\left[50\;\left[m\right],10\;\left[m/s\right],80\;\left[m\right],20\;\left[m/s\right]\;\right]}^{T} \end{array}$$

(103) $$\begin{array}{cc} P_{0} & =\left[\begin{array}{cccc} 1\;[m^{2}] & 0 & 0 & 0\\ 0 & 1\;[m^{2}/s^{2}] & 0 & 0\\ 0 & 0 & 1\;[m^{2}] & 0\\ 0 & 0 & 0 & 1\;[m^{2}/s^{2}] \end{array}\right]. \end{array}$$

Monte Carlo simulations have been carried out with 100 independent trials. The CIFF of Section 4.1 and the proposed VBST-CIFF of Section 4.2 are compared in the above described multi-sensor target tracking scenario. Specifically, in CIFF, *Q* and *R* are fixed to $Q=Q_{k-1}$ for both sensors, $R=\Lambda_{k,1}$ for sensor 1, and $R=\Lambda_{k,2}$ for sensor 2. Conversely, in the proposed VBST-CIFF, $Q=Q_{k-1}$, while the scale matrix of each sensor as well as other parameters are adaptively estimated. The same values for the simulation time *t*, sampling interval *T*, forgetting factor τ, number of VB iterations *N*, as well as initial parameters of the scale matrix (δ, Δ), initial parameters of the DOF parameter (ϕ, Φ) of the previous single-sensor case-study of Section 5.1 have been adopted.

**Figure 7:**
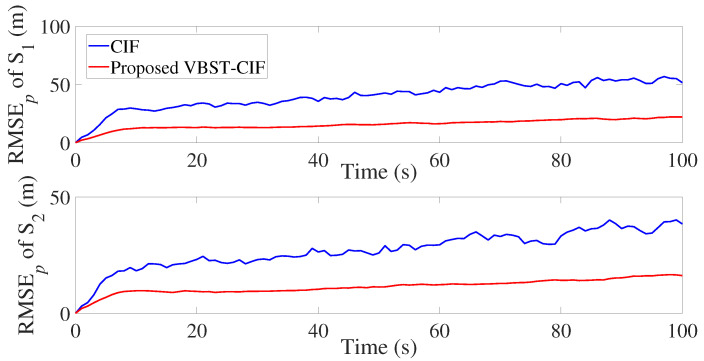
Position RMSE of different sensors in multi-sensor target tracking.

**Figure 8:**
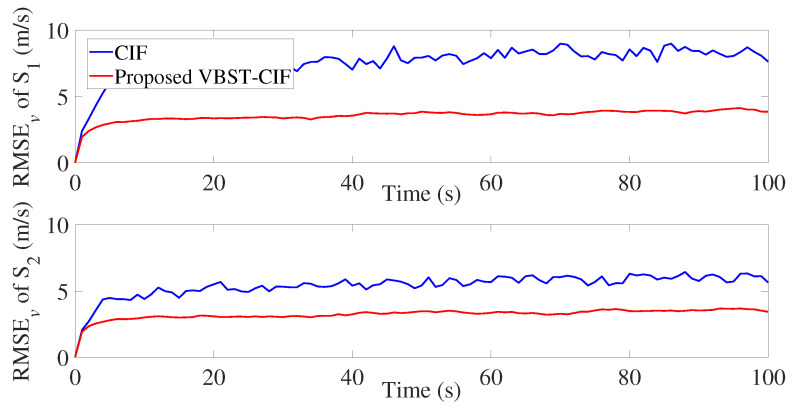
Velocity RMSE of different sensors in multi-sensor target tracking.

**Figure 9:**
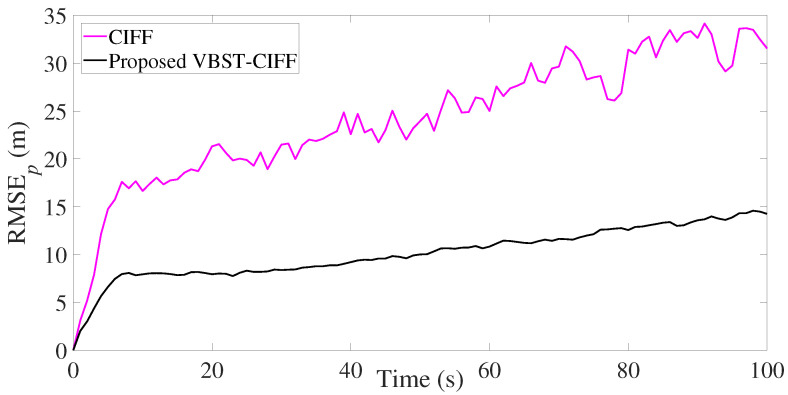
Position RMSE of fusion in multi-sensor target tracking.

**Figure 10:**
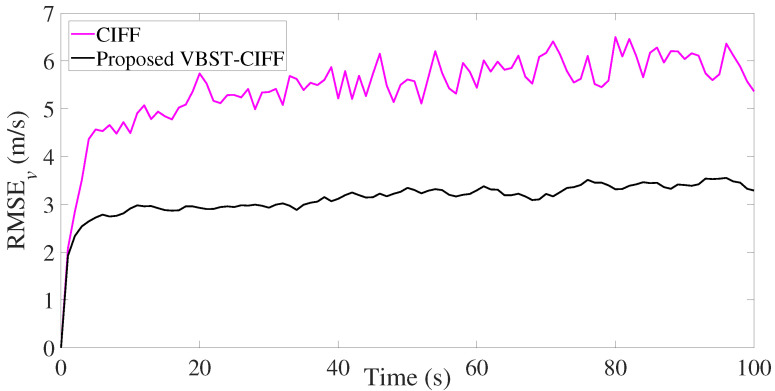
Velocity RMSE of fusion in multi-sensor target tracking.

The results are presented in Figure 7, Figure 8, Figure 9 and Figure 10 and Table 2. It is clear from Figure 7 and Figure 8 that the proposed VBST-CIF provides, compared to conventional CIF, smaller RMSEs in both position and velocity for each local sensor during the whole simulation time. Figure 10 shows that the proposed VBST-CIFF outperforms the conventional CIFF, at the fusion center during the whole simulation time. Furthermore, it can be seen that in Table 2 the proposed VBST-CIFF provides an MRMSE improvement of 74% in position and 58% in velocity compared to conventional CIF with sensor 1, as well as an improvement of 62% in position and 42% in velocity compared to CIF with sensor 2. Moreover, the proposed VBST-CIFF provides an MRMSE improvement of 58% in position and 42% in velocity compared to CIFF.

### 5.3. Experimental Case-Study

**Figure 11:**
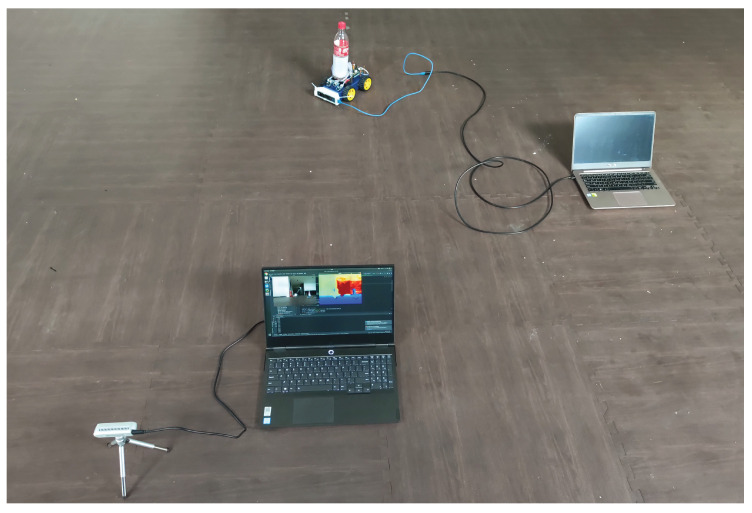
Experimental setup.

Let us consider the experimental setup of Figure 11. A model-car moves along a straight line with a nearly constant velocity starting from initial position (1018,2619)[mm]. A depth camera (Camera 1, Intel RealSense Depth Camera D435i depth sensor) located at (0,0) measures its distance from the moving car, while another depth camera (Camera 2, Intel RealSense Tracking Camera T265), located onboard the car can get the location and azimuth angle of the car with respect to Camera 1. Please notice that the performance of Camera 1 is highly affected by the unstable Tiny YOLOv3 detector. Thus, the measurement function is the same as in (93) with a measurement noise of unknown heavy-tailed distribution. The total sampling samples are 363, with a sampling interval of T=1/60[s]. Moreover, initial values of the model-car are selected as:

(104) $$\begin{array}{cc} x_{0} & ={\left[1018\;\left[\mathrm{mm}\right],\;2619\;\left[\mathrm{mm}\right],\;228.76\;\left[\mathrm{mm}/s\right],\;311.90\;\left[\mathrm{mm}/s\right]\;\right]}^{T} \end{array}$$

(105) $$\begin{array}{cc} P_{0} & =\left[\begin{array}{cccc} 1\;[{\mathrm{mm}}^{2}] & 0 & 0 & 0\\ 0 & 1\;[{\mathrm{mm}}^{2}] & 0 & 0\\ 0 & 0 & 1\;[{\mathrm{mm}}^{2}/s^{2}] & 0\\ 0 & 0 & 0 & 1\;[{\mathrm{mm}}^{2}/s^{2}] \end{array}\right]. \end{array}$$

**Figure 12:**
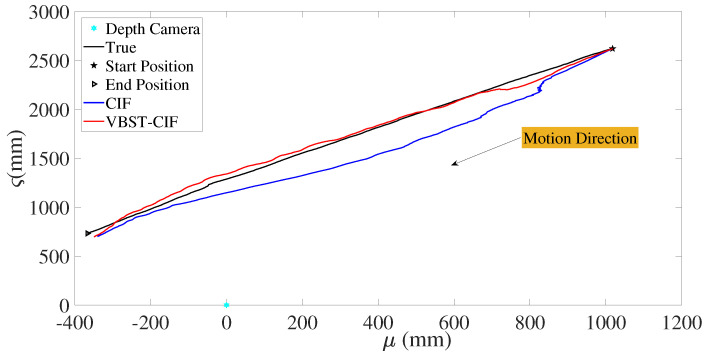
Car trajectory.

**Figure 13:**
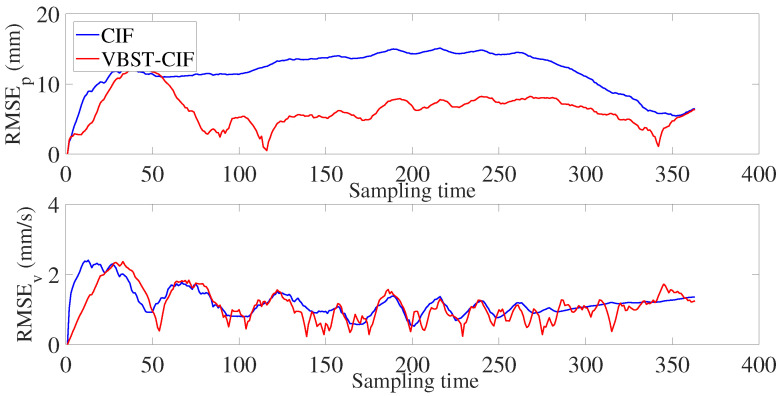
Position and velocity RMSEs.

Let us consider the experimental results reported in Figure 12 and Figure 13 and Table 3. Figure 12 shows the true and estimated car trajectories, from which it can be seen that the proposed VBST-CIF can track the car more accurately. Position and velocity RMSEs of both conventional CIF and proposed VBST-CIF are reported in Figure 13. Specifically, the proposed VBST-CIF provides an improvement of 46.24% in position MRMSE and of 0.08% in velocity MRMSE, with respect to conventional CIF, thus demonstratiung the effectiveness and superiority of the proposed VBST-CIF.

## 6. Conclusions

This paper focused on state estimation and information fusion in multi-sensor nonlinear systems with heavy-tailed measurement noise. An adaptive variational Bayes Student’s t cubature information filter (VBST-CIF) algorithm was first designed for a nonlinear single-sensor system based on the cubature information filter (CIF) framework, wherein the Student’s t (ST) distribution was utilized to model the heavy-tailed measurement noise, and the variational Bayes (VB) method along with the spherical-radial cubature (SCR) rule were exploited in order to jointly estimate the system state as well as noise statistics. The VBST-CIF facilitated multi-sensor fusion, allowing to derive a VBST cubature information feedback fusion (VBST-CIFF) algorithm for nonlinear multi-sensor systems with heavy-tailed measurement noise. The filtering accuracy of the proposed algorithms was assessed in single-sensor and multi-sensor target tracking scenarios. Simulation and experimental results demonstrated that the proposed VBST-CIF/VBST-CIFF outperformed conventional CIF/CIFF algorithms. Future work will focus on consensus filtering for multi-agent systems with heavy-tailed process and measurement noise.

## Figures and Tables

**Table 1:** Mean-root-mean-square-error (MRMSE) in single-sensor target tracking.

Filter	${\mathrm{RMSE}}_{p}$	${\mathrm{RMSE}}_{v}$	${\mathrm{RMSE}}_{\omega }$
CIF	5.2364	1.8646	0.0518
VBST-CIF	2.3052	0.9740	0.0412

**Table 2:** MRMSEs in multi-sensor target tracking.

Filter	Sensor	${\mathrm{RMSE}}_{p}$	${\mathrm{RMSE}}_{v}$
CIF	1	40.1874	7.5373
CIF	2	27.1914	5.4180
CIFF	Fusion	24.1697	5.4225
VBST-CIF	1	15.5891	3.5502
VBST-CIF	2	11.4030	3.2353
VBST-CIFF	Fusion	10.1193	3.1117

**Table 3:** MRMSEs in the experimental case study.

Filter	${\mathrm{RMSE}}_{p}$ [mm]	${\mathrm{RMSE}}_{v}$ [mm/s]
CIF	11.7397	1.1985
VBST-CIF	6.3111	1.0991
